# Genome-Wide Analysis of Copy Number Variation in Vietnamese Local Chickens

**DOI:** 10.3390/ani16071085

**Published:** 2026-04-01

**Authors:** Thuy Thi-Dieu Nguyen, Ana Tzvetkova, Mai Thi-Dieu Bui, Vo-Anh-Khoa Do, Thuy Thi-Ngoc Dinh, Phuong Thanh Nguyen, Andreas Walter Kuss, Mauro Penasa, Filippo Cendron

**Affiliations:** 1 Department of Animal Biotechnology, Institute of Biology, Vietnam Academy of Science and Technology, 18 Hoang Quoc Viet, Nghia Do, Hanoi 11307, Vietnam; thuy81ibt@gmail.com (T.T.-N.D.); ntphuong2901@gmail.com (P.T.N.); 2 Interfaculty Institute for Genetics and Functional Genomics, University of Greifswald, Felix-Hausdorf-Str. 8, 17489 Greifswald, Germany; ana.tzvetkova@uni-greifswald.de (A.T.); kussa@uni-greifswald.de (A.W.K.); 3 Department for Promoting Business Cooperation, Faculty of Agronomy, Vietnam National University of Forestry, Dongnai Campus, Trang Bom 76306, Vietnam; dieumai2804@gmail.com (M.T.-D.B.); dvakhoa@gmail.com (V.-A.-K.D.); 4 Department of Agronomy, Food, Natural Resources, Animals and Environment, University of Padova, 35020 Legnaro, Italy; mauro.penasa@unipd.it

**Keywords:** copy number variant, Vietnamese indigenous chicken, Dong Tao, NGS, genomic variation

## Abstract

Copy number variants (CNVs) are large-scale genomic alterations that contribute substantially to genetic diversity and may influence phenotypic variation in livestock. This study investigated the genome-wide CNV landscape of three Vietnamese indigenous chicken breeds. Whole-genome sequencing on the Illumina platform (3–5× coverage) was performed on 24 individuals from Dong Tao (DT), Cay Cum (CC), and Ri (RI) breeds. A total of 1743 CNVs were detected, clustering into 315 copy number variation regions (CNVRs). Most CNVRs were rare, with 31.7% present in only one animal among breeds. Across the genome, 122 unique CNVRs were distributed over 28 chromosomes, predominantly the first five. Losses were the most frequent type (45.9%), followed by gains (39.3%), and mixed events (14.8%). Within these CNVRs, 3633 genes were identified. In DT and RI, CNVR-embedded genes included several candidates, potentially related to adaptability, development, and phenotypic diversification. Notably, DT harbored genes such as *EGLN1*, *OASL*, *GPX1*, *DUOX1/DUOXA2* (adaptation, stress/immune response) and *LRP4*, *ZIC1*, *ZIC4*, *JARID2*, *KMT2C*, *OGN*, *OMD*, and *PLOD2* (developmental and skeletal traits), whereas in RI they included genes such as *CACNA1S*, *CALCR*, *CAPN3*, and *MAPK13/MAPK14*, which may contribute to muscle, bone, and physiological regulation. Functional enrichment analysis revealed numerous genes and Quantitative Trait Loci (QTLs) associated with metabolic, developmental, and immune-related pathways. This study provides the first comprehensive genome-wide CNV profile of Vietnamese indigenous chickens and offers a valuable genomic resource for investigating the genetic basis of breed-specific and adaptive phenotypes.

## Simple Summary

This study aimed to characterise the genome-wide distribution of copy number variants (CNVs) and copy number variation regions (CNVRs) in Vietnamese indigenous chickens. A total of 24 individuals from Dong Tao (DT), Cay Cum (CC), and Ri (RI) breeds were sampled, and whole-genome sequenced on an Illumina platform at 3–5× coverage. Across the three breeds, 1743 CNVs were identified and clustered into 122 unique CNVRs. Functional enrichment analyses revealed numerous genes and Quantitative Trait Loci overlapping with these CNVRs, associated with a variety of biological functions and pathways. Overall, this study provides the first comprehensive genome-wide overview of CNVs and CNVRs in Vietnamese local chickens and establishes a foundation for future research into the genetic characterisation of local Vietnamese breeds.

## 1. Introduction

Copy number variation is a type of genetic variation, representing a deviation from the normal diploid state, caused by the loss or gain of genomic regions. Copy number variants (CNVs) are widely distributed across the genome in various sizes, ranging from one kilobase to several million base pairs (Mb), and cause differences in copy numbers among individuals within a population [1,2]. These variations are formed through structural rearrangements such as inversion, translocation, duplication or deletion within copy number variation regions (CNVRs) [3,4,5], and contribute to phenotypic diversity [6,7]. Constructing copy number variation profiles for chicken breeds not only clarifies evolutionary mechanisms and domestication processes but also provides a valuable database for molecular breeding and the improvement of indigenous chicken populations [8,9]. Copy number variation analysis methods, including array-based comparative genomic hybridization [10,11,12], high-density SNP chips [9,13,14,15,16,17], and next-generation sequencing (NGS) [5,8,18,19,20,21], have significantly enhanced the ability to detect CNVs with high resolution, thereby supporting the construction of comprehensive copy number variation maps [17]. Several studies have shown that CNVs are diversely distributed across chromosomes and are closely associated with important traits in local chickens, such as productivity, morphology, and disease resistance [7,20,22].

Recent large-scale studies have affirmed the significant achievements of research on CNVs in elucidating the genetic basis of important economic traits in chickens. Cendron et al. [17] identified hundreds of CNVRs that show significant overlap with QTLs influencing productivity, egg quality, and chicken adaptability, revealing potential applications in breeding programs. Another study pinpointed numerous functional genes adjacent to CNVRs associated with economically important traits [15]. Further studies have revealed significant associations between CNVs clustered on specific chromosomes and bone morphological characteristics, while also identifying genes regulating bone growth in chickens [5,22]. Another achievement in research on CNVs includes the confirmation of the Major Histocompatibility Complex (MHC) region and the detection of CNVs affecting immune-related genes, such as those conferring heat stress tolerance and immune response [17] as well as candidate genes specifically linked to disease resistance [13,20,23]. For phenotypic and meat quality traits, the studies on CNVs have provided insights into the genetics of feather color [13,23], uncovered CNVRs that affect metabolism and fat accumulation [12,16], and revealed correlations with body weight and weight gain [9].

Indigenous chickens play a key role in the agricultural economy and nation-specific culture due to their high adaptability to local environmental conditions, efficient utilization of local feed resources, and natural disease resistance. Many local chicken breeds harbour valuable traits, including superior meat flavour, specialty eggs, and unique phenotypes, which contribute to product diversification and serve as an invaluable genetic resource for breeding and conservation programs [24]. On the other hand, they also possess certain undesirable characteristics, such as slow growth, late maturity, and low productivity. The Vietnamese Dong Tao (DT) chicken is characterized by a large body size and massive dark red shanks, which are comparatively thick, oversized, and covered in layered horny scales. The distinguishing morphological feature of the Cay Cum (CC) chicken breed is its rumpless phenotype. The CC and Ri (RI) breeds are both characterized by smaller body size and more slender legs than DT, as well as a remarkable adaptability to various geographical and climatic conditions in Vietnam.

This study aims to analyse the distribution of CNVs and CNVRs across the entire genome of the DT, CC, and RI breeds. The CNV/CNVR profiles could be linked to potential candidate genes/QTLs, which are available in the Animal QTLdb [https://www.animalgenome.org/cgi-bin/QTLdb/index (accessed on 18 February 2026)], thereby suggesting potential pathways associated with important traits and specific phenotypes. As such, they represent an important resource for the sustainable conservation and breeding programs of Vietnamese local poultry.

## 2. Materials and Methods

### 2.1. Sampling

Whole blood samples were taken from the wing vein of DT (*n* = 12), CC (*n* = 6), and RI (*n* = 6) animals and kept at 4 °C for subsequent DNA extraction. All individuals were selected based on the presence of typical characteristics of the respective breeds. DT chickens were sampled at the Dong Tao commune (Hung Yen province), CC chickens derived from the provinces Cao Bang and Ha Giang, and RI chickens from the Ri breeding station in Lien Ninh, Hanoi, Vietnam.

### 2.2. DNA Extraction, Library Preparation, and Sequencing

Genomic DNA was extracted using a standard Phenol/Chloroform-based protocol. The quantity and quality of DNA were checked by agarose gel electrophoresis and Qubit (Thermo Fisher Scientific Inc., Waltham, MA, USA) analysis. Genomic DNA was enzymatically digested into 150–250 bp fragments and used to create paired-end libraries by NEBNext^®^ Ultra™ II DNA Library Prep Kit for Illumina^®^ (Code #E7103S/L) sequencing (Illumina Inc., San Diego, CA, USA). Libraries were quality checked using a Bioanalyzer device (Agilent, Santa Clara, CA, USA) and real-time quantitative polymerase chain reaction (qPCR), denatured, diluted to a concentration of 20 pM, and used for NGS. Sequencing was carried out at the Institute of Biology of the Vietnam Academy of Science and Technology, Hanoi, Vietnam, using a NextSeq™550Dx High Output Reagent Kitv2.5 (300 Cycles) (Illumina Inc., San Diego, CA, USA) on an Illumina NextSeq 550 Sequencer (Illumina Inc., San Diego, CA, USA) with 3–5-fold coverage. The raw reads were first checked with FastQC and subsequently mapped to the reference genome employing BWA v0.7.17 software [25]. As a reference, we used the latest chicken genome version, GRCg7b [https://www.ncbi.nlm.nih.gov/datasets/genome/GCF_016699485.2/ (accessed on 25 January 2026)]. For calculating the average coverage, the Mosdepth tool was applied [26].

### 2.3. CNV Calling and CNVRs with Overlapping Known QTLs

CNVs were identified independently using FREEC v11.6 and CNVpytor v1.3.1. In both pipelines, the genome was partitioned into fixed-size, non-overlapping bins. Read depth was quantified per bin, and counts were normalized for GC-content and mappability bias, using a GEM-derived mappability track for the chicken reference genome. CNV calls were then generated by segmentation of normalized read-depth profiles into regions with homogeneous copy-number states [27]. The two call sets were compared by genomic intersection to assess cross-caller concordance. Therefore, rather than defining the final CNV dataset solely on cross-caller overlap, we retained CNVs that met stringent statistical and quality criteria, including length/support thresholds based on the FREEC binning strategy (bin size = 50 kb), CNVpytor e-val1 < 0.01, q0 < 0.5, and exclusion of calls overlapping poorly mappable or assembly-gap-rich regions (e.g., mean mappability < 0.5) [28].

To determine the functional relevance of the detected CNVs, we merged overlapping or adjacent CNVs across individuals to define CNVRs. The genomic intersections between CNVRs and annotated genes, as well as between CNVRs and known QTLs, were computed using BEDTools (version 2.31.0) in a Linux environment. BEDTools intersect was used with default parameters to identify (i) CNVRs overlapping gene coordinates from the NCBI annotation and (ii) CNVRs overlapping QTLs retrieved from the Animal QTLdb [https://www.animalgenome.org/cgi-bin/QTLdb/index (accessed on 18 February 2026)]. Only overlaps of at least 1 bp were considered significant, and all genomic coordinates were harmonized to the same reference assembly prior to analysis. Chromosome-wise analyses were performed considering the 28 autosomes retained in the filtered dataset, based on the chicken reference assembly [17].

### 2.4. CNV Validation

qPCR was carried out to validate the CNVRs, using a QuantStudio 5 System (Applied Biosystems, Foster, CA, USA). Five CNVRs were randomly selected and specific primers for each region were designed based on BLAST-Primer and checked by the PrimerStat tool (Table S1). For each CNVR tested, three reference samples (2n) and three different testing animals were selected for qPCR. Firstly, the amount of DNA of each sample was estimated by a NanoDrop 1000 Uv (Thermo Fisher Scientific, Waltham, MA, USA) and diluted to a final concentration of 25 ng/uL. qPCR was done using Luminaris Higreen Low ROX qPCR Master Mix (Thermo Fisher Scientific, Waltham, MA, USA). The GADPH gene was used as a normalization control for the samples. A final 10 μL reaction of qPCR consisted of 1× Luminaris Higreen Low ROX qPCR Master Mix, 50 nm forward and reverse primers and 50 ng of genomic DNA. The qPCR thermal cycling conditions included three stages: the hold stage with 50 °C for 2 min and initial denaturation at 95 °C for 10 min; the PCR stage of 40 cycles (denaturation at 95 °C for 15 s, annealing at 60 °C for 15 s and extension at 72 °C for 30 s); and the melting curve stage with 60 °C for 15 s and 95 °C for 1 s. Three replicates were performed for each sample, and the mean cycle threshold (Ct) values were used for further analysis. The copy number difference in the target CNVRs in the experimental samples was determined using the 2^−ΔΔCt^ method [29]. For qPCR primer efficiency, the standard curve of each primer was established with serial dilution series, starting with an undiluted DNA sample, and then a 1:10 dilution for creating a standard curve with 5-point dilutions (Table S2). The slope of the regression between the log value of sample concentration and average Ct value was calculated. Finally, the primer efficiency was calculated as follows:

$$E=-1+10^{\left(\frac{-1}{slope}\right)}$$

## 3. Results

### 3.1. Identification of CNVs/CNVRs

#### 3.1.1. By Breed

A total of 1743 CNVRs were identified: 855 for DT, 430 for RI, and 458 for CC. In total, 315 CNVRs were detected among the three breeds, with the largest number (*n* = 129) in DT chickens (58 losses, 48 gains, and 23 mixed), followed by CC (*n* = 106; 44 losses, 45 gains, and 17 mixed), and RI (*n* = 80; 56 losses, 6 gains, and 18 mixed). Genome coverage by CNVRs was 8.6% in DT, 7.6% in CC, and 4.7% in RI. The CNVR length was highest (11,650,000 bp) and lowest (11,339 bp) in CC (Table 1).

#### 3.1.2. CNVRs Frequency and Overlap Patterns Across Breeds

**Figure 1:**
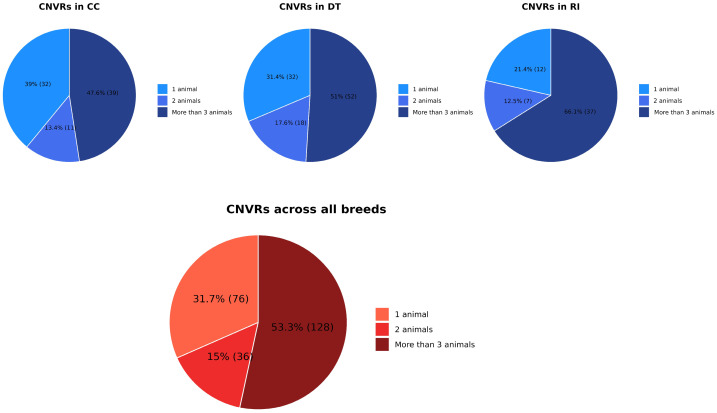
Copy number variation regions (CNVRs) shared among animals within breeds and across breeds (CC = Cay Cum; DT = Dong Tao; RI = Ri). Values in parentheses indicate the number of CNVRs.

Across the three breeds, most CNVRs were detected in only one individual, although the proportions varied among populations. In CC, 39% of CNVRs were single-animal events, followed by 13.4% detected in two animals, and 47.8% in more than two animals. In DT, 31.4% of CNVRs occurred in a single individual, 17.6% in two individuals, and 51% in more than two individuals. In RI, 21.4% of CNVRs were identified in one animal, 12.5% in two animals, and 66.1% in more than two animals. When all breeds were considered together, 31.7% of CNVRs were observed in a single individual, 15% were shared by two individuals, and 53.3% were present in more than two individuals (Figure 1). The frequency of CNVRs appearing in more than two individuals predominates in all three breeds. Most CNVRs were detected in a single breed, indicating a breed-specific component, while only a few CNVRs were observed in all individuals of a given breed (i.e., one in DT and two each in CC and RI), suggesting recurrent within-breed structural variants rather than strictly breed-exclusive fixed events (Table S3).

**Figure 2:**
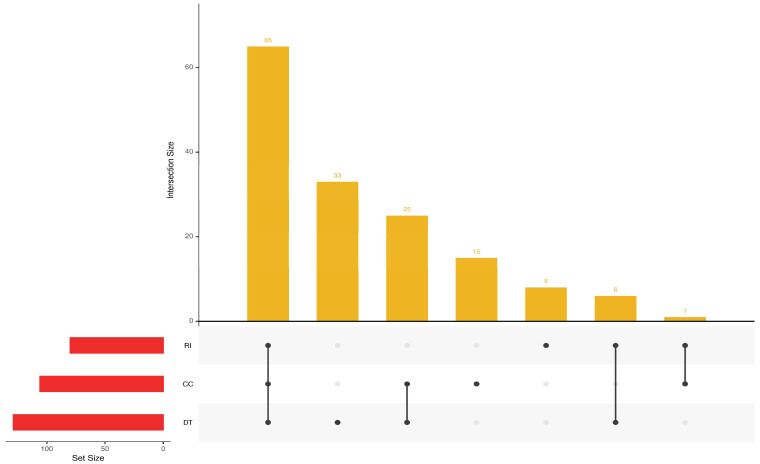
UpSet plot showing shared and unique copy number variation regions (CNVRs) among the three Vietnamese indigenous chicken breeds: Dong Tao (DT), Cay Cum (CC), and Ri (RI). The horizontal red bars on the left represent the total number of CNVRs identified in each breed. The vertical yellow bars indicate the size of each intersection set, corresponding to the number of CNVRs shared among specific combinations of breeds, as illustrated by the connected black dots below each bar. Single black dots represent CNVRs unique to a single breed and connected dots represent CNVRs shared between two or all three breeds.

The UpSet plot (Figure 2) summarizes the distribution of the 122 non-redundant CNVRs across breeds. The set sizes indicate the number of CNVRs present in each breed, while the intersection bars represent CNVRs shared between breeds. Because each CNVR can occur in one or more breeds, the intersection counts do not sum to the total number of CNVRs. Among the shared sets, the largest pairwise intersection was observed between CC and DT, which shared 25 CNVRs. RI and DT shared six CNVRs, and RI and CC shared only one CNVR. One intersection consisting of 65 CNVRs was shared among all three breeds, accounting for the largest proportion of shared CNVRs. The barplot on the left highlights differences in the total CNVR counts per breed, with DT having the highest overall count, followed by CC and RI. The combination of unique and shared sets shown in the UpSet matrix demonstrates that the CNVR landscape is dominated by shared CNVRs across all three breeds (Figure 2).

#### 3.1.3. By Chromosome

**Figure 3:**
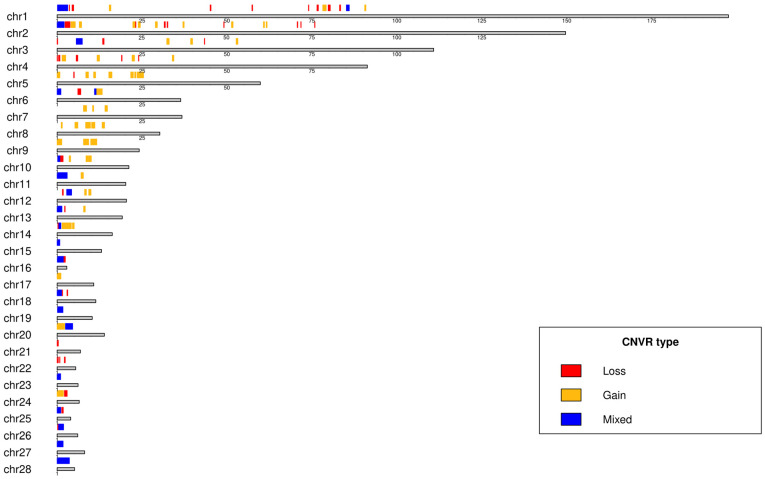
Idiogram of the 28 chicken chromosomes showing copy number variation regions (CNVRs) identified across breeds.

Figure 3 depicts the distribution map of unique, non-duplicated CNVRs by type on each chromosome, and a detailed overview is fully presented in Table 2. A total of 122 unique, non-duplicated CNVRs were identified and showed an uneven distribution across the 28 chromosomes, being predominantly concentrated on the first five chromosomes. Regarding the type of variation, the loss type was dominant (56 CNVRs, 45.9%), followed by gain (48 CNVRs, 39.3%), and mixed (18 CNVRs, 14.8%). The proportion of CNVRs relative to chromosome length ranged from 3.8% (chromosome 3) to 82.0% (chromosome 16). A notable finding was the highest coverage proportion of CNVRs on chromosome 16, even though it is the smallest chromosome.

#### 3.1.4. Validation of CNVRs by qPCR

The qPCR results show a validation rate of 80%, with four out of five primer pairs confirming the presence of CNVRs (Table S2). The results confirm the predicted CNVs identified by FREEC. The primer efficiency in this study ranged from 94.8 to 104.9%, which is acceptable for reliable qPCR.

### 3.2. CNVRs Overlapping Genes/QTLs

A total of 3633 genes were identified across the three Vietnamese chicken breeds, with some genes shared among all breeds (Table S4). The DT breed, known for its markedly enlarged shanks, had the highest number of genes (*n* = 3306) inside the CNVRs, followed by CC (*n* = 3080) and RI (*n* = 2048). This enrichment supports a broader structural genomic variability in DT, which may contribute to breed-specific phenotypic traits, including limb morphology. In DT, several CNVRs overlapped genes with plausible roles in developmental regulation, skeletal/mesenchymal traits, and extracellular matrix organization, including *LRP4* (bone homeostasis and skeletal signaling), *ZIC1* and *ZIC4* (developmental transcription factors involved in embryonic patterning and morphogenesis), *JARID2* and *KMT2C* (epigenetic/chromatin regulators of developmental gene expression), and *PLOD2*, *OGN*, and *OMD* (collagen modification and extracellular matrix components associated with connective tissue and bone-related processes). *ACTA1*, *BGN*, and *CREB3L2* further support potential effects on muscle/structural function, matrix organization, and tissue remodeling. In addition, CNVRs in the DT breed included genes linked to physiological adaptation and stress/immune-related responses, such as *EGLN1* (cellular oxygen sensing/hypoxia signaling), *GPX1* (antioxidant defense against oxidative stress), *OASL* (innate antiviral immune response), and *DUOX1/DUOXA2* (reactive oxygen species generation and epithelial host defense). Altogether, these findings indicate that CNVRs in DT affect a broad set of genes with plausible relevance to developmental, structural, and adaptive phenotypes.

Integration of the CNV dataset with the QTL database (Table S5) identified a total of 2901 CNVR–QTL overlaps across the three breeds. DT showed the highest number of CNVRs intersecting at least one QTL (*n* = 75), followed by CC (*n* = 65), and RI (*n* = 42). In terms of total overlap events, DT (*n* = 1073) and CC (*n* = 1121) displayed comparable numbers, whereas RI showed fewer overlaps overall (*n* = 707). Several overlapping QTL annotations were related to skeletal and morphometric traits (e.g., shank/tibia/bone-related traits), which are potentially relevant to the DT phenotype. However, many QTL terms were broader categories (e.g., body weight gain, growth rate, carcass traits), and QTL intervals can span large genomic regions.

### 3.3. Gene Ontology Terms

**Figure 4:**
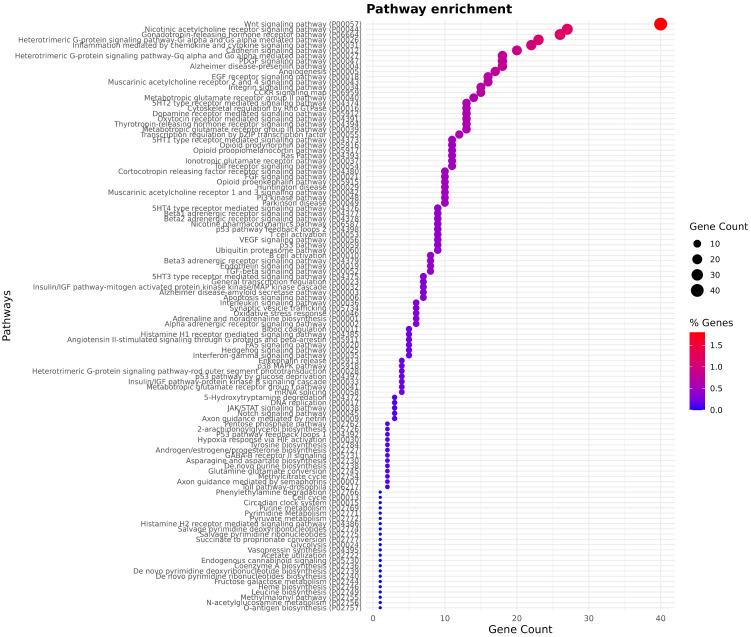
Pathway enrichment analysis of genes located within copy number variation regions. Dot size indicates the number of genes per pathway and color reflects the percentage of genes within each pathway.

Pathway enrichment analysis showed that CNVR-overlapping genes were distributed across a broad set of biological pathways (Figure 4). Most enriched pathways were represented by relatively low gene counts, indicating that the majority of functional categories included a limited number of CNVR-associated genes. However, a smaller subset of pathways displayed higher gene counts and higher percentages of mapped genes. The enriched pathways were predominantly related to signal transduction, with strong representation of receptor-mediated and G protein-coupled signaling pathways, including heterotrimeric G-protein signaling, cholinergic (nicotinic and muscarinic) signaling, glutamatergic signaling, and other neurotransmitter-related pathways. Additional enriched categories included developmental signaling pathways (e.g., WNT signaling) and hormone/receptor-mediated signaling pathways. Overall, the enrichment profile suggests that CNVR-associated genes are mainly involved in signaling networks, with many pathways showing modest but specific gene representation.

**Figure 5:**
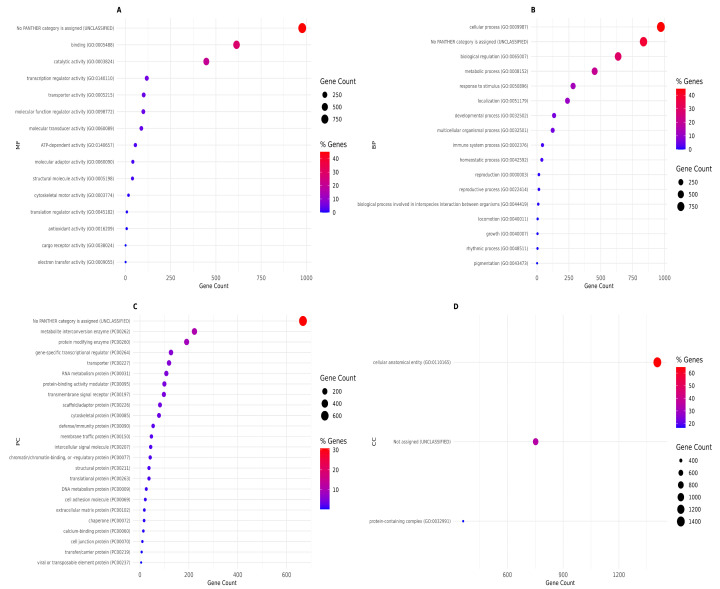
Gene Ontology (GO) enrichment analysis of genes located within copy number variation regions. Panels show enriched GO terms for (**A**) Molecular Function (MF), (**B**) Biological Process (BP), (**C**) Protein Class (PC), and (**D**) Cellular Component (CC). Dot size represents gene count per term and color indicates the percentage of genes contributing to each GO term.

Figure 5 depicts the Gene Ontology enrichment results for the genes located within CNVRs. In the Molecular Function category (Figure 5A), most of the genes fell into broad functional groups such as binding activity, catalytic activity, transcription regulator activity, and transporter activity. In the Biological Process category (Figure 5B), the most represented processes were cellular and metabolic processes, biological regulation, and response to stimulus. Homeostasis and immune system processes were also enriched. Protein Class enrichment (Figure 5C) provided a more detailed view of the types of proteins encoded by CNVR-associated genes. Many belonged to metabolite interconversion enzymes, protein-modifying enzymes, transcriptional regulators, and various transporter families. The Cellular Component category (Figure 5D) showed a more limited number of enriched terms, mainly highlighting genes associated with cellular anatomical entities and protein-containing complexes.

### 3.4. Cluster-Tree

**Figure 6:**
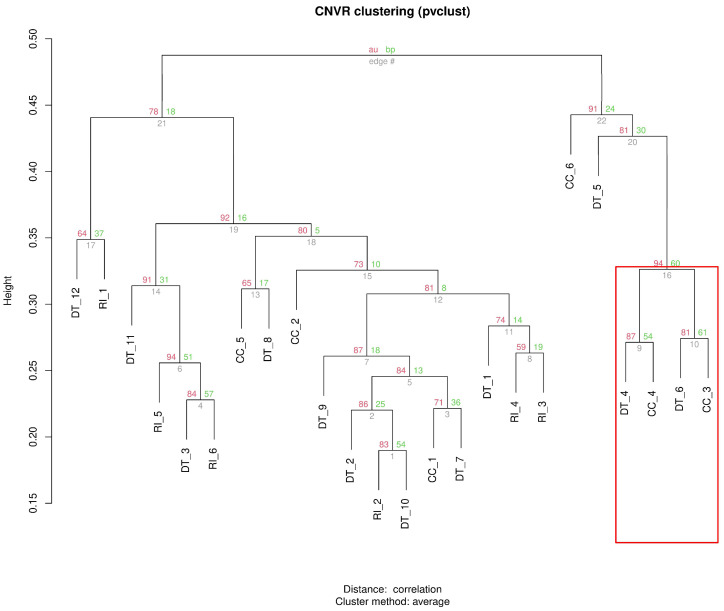
Hierarchical clustering of individuals based on copy number variation region (CNVR) profiles. Approximately unbiased (AU) *p*-values (%) indicate cluster support, highlighting genomic similarities and differences among the three chicken breeds. The cluster dendrogram reports AU-P (approximately unbiased *p*-value; dark grey color) and BP-*p* values (bootstrap probability *p*-value; grey color) among breeds (%). The edge is reported in light grey color. Breeds with high copy number variant similarities are in the red box.

Figure 6 shows the hierarchical clustering of individuals based on CNVR profiles, with branch support assessed using approximately unbiased (AU) *p*-values calculated by pvclust. Overall, the dendrogram highlights substantial variability both within and between the three indigenous chicken breeds, with several individuals from different breeds clustering together. In particular, DT individuals do not form a single breed-specific cluster but are distributed across multiple branches of the tree. A well-supported mixed subcluster is observed in the highlighted region, where DT_4 clusters with CC_8 and DT_6 clusters with CC_3; these two pairs then group together with high support (AU = 94%). Additional mixed groupings are also present elsewhere in the dendrogram, indicating partial overlap in CNVR profiles among breeds. RI and CC individuals also show an intermixed pattern, and several internal nodes display moderate or low AU support, suggesting that some relationships are less stable.

## 4. Discussion

### 4.1. Investigation of CNVs and CNVRs

The 1743 CNVs and 315 unique CNVRs detected in Vietnamese indigenous chickens in the current study were much lower than those reported in studies on native chickens from China [18], Mexico [13], and Italy [17] (Table S6), likely due to variation in the number of breeds and individuals analysed, as well as differences in sequencing depth and analytical methods among studies.

The combination of a high number of breed-restricted CNVRs and a small subset of highly recurrent CNVRs within breeds supports the presence of structured CNV variation among Vietnamese local chickens, with DT showing the broadest CNVR landscape. Indeed, DT showed the highest number of CNVRs, followed by CC and RI, suggesting substantial variability in the extent of genomic structural rearrangements among these populations. Such differences may reflect distinct demographic histories, varying levels of selection pressure, or breed-specific adaptation processes [17]. Breed-level variation was also evident in CNVR length profiles and genome coverage, with DT exhibiting both the largest number of events and the highest cumulative genomic span. These findings are consistent with the phenotypic distinctiveness of DT, particularly its hypertrophied shanks, and may point to a more complex structural genomic landscape in this breed. In contrast, RI had fewer CNVRs and lower genome coverage, suggesting either reduced structural variability or a more conserved genome relative to the other breeds. Patterns of CNVRs sharing further support this differentiation. The substantial overlap between DT and CC suggests closer genomic similarity or partially shared ancestry between these two breeds, whereas the limited intersection involving RI is consistent with a more distinct genomic background and potentially different selective pressures or demographic history (Figure 2). Collectively, these patterns highlight the heterogeneous nature of structural variation in Vietnamese native chickens and the importance of CNVs and CNVRs as contributors to breed differentiation and unique phenotypic features.

The pattern where the majority of CNVRs were detected in only one animal (Figure 1) indicates a predominance of low-frequency or individual-specific structural variants. This pattern is frequently observed in local or unselected chicken populations, where heterogeneous breeding histories lead to high genomic variability. For example, in Mexican chickens, 82.9% of CNVRs were singletons [13], and in Chinese chicken populations, 68.8% of CNVRs were detected in only one individual [18]. The overall distribution highlights the substantial genomic diversity present in Vietnamese indigenous breeds, reflecting their adaptation to diverse local environments and the limited impact of strong directional selection typically observed in commercial poultry. The higher number of multi-individual CNVRs in DT compared with CC and RI may be associated with its distinct morphology, particularly the massive shank phenotype, which could be influenced by structural variants shared across individuals due to breeding preferences or historical selection.

Taken together, the results demonstrate that CNVR patterns differ considerably among the three breeds, with DT and CC showing closer genomic relationships and RI exhibiting a more unique structural variant profile. The prevalence of rare CNVRs further underscores the genetic richness of these indigenous chickens and supports their value as important reservoirs of unique genomic diversity. Such CNVRs may contribute to adaptive traits, local resilience, and distinctive phenotypes, and they represent promising targets for future functional characterization and conservation-oriented breeding strategies.

Notably, CNVRs were detected on chromosome 16 (Table 2), which is consistent with several previous studies reporting that this chromosome, despite being the smallest, frequently harbors a high density of CNVs [13,17,18]. This finding is biologically plausible given the functional importance of chromosome 16, which contains key immune-related genes, including the MHC, a genomic region known for extensive natural variation driven by pathogen-mediated selective pressure. The presence of CNVRs in this region further supports the relevance of structural variation in genomic regions potentially involved in immune response and adaptation.

### 4.2. Genes and QTLs Within CNVRs

The CNVR analysis supports the view that structural variation may contribute to within- and between-breed genomic heterogeneity, while also highlighting the need for cautious biological interpretation. In our dataset, the distribution of CNVR-overlapping genes across breeds (Table S4) suggests that CNVs capture a broad and functionally diverse fraction of the genome, rather than a small set of recurrent loci with obvious and direct phenotypic effects. In particular, the higher number of CNVR-embedded genes observed in DT may indicate a comparatively broader structural genomic variability in this breed. This is more appropriately viewed as a starting point for hypothesis generation, especially in relation to developmental, structural, physiological, and immune-related processes that may contribute to breed-specific phenotypic profiles.

The genes identified in CNVRs of DT point to a more distributed architecture involving multiple biological systems. This interpretation is more consistent with current understanding of complex morphological traits, which are often polygenic and influenced by combinations of regulatory, extracellular matrix (ECM), signaling, and physiological pathways rather than single gene clusters alone. In this context, CNVs may contribute by altering dosage, local chromatin context, or regulatory landscapes, but these mechanisms remain speculative in the absence of expression or functional validation. More broadly, CNVs are recognized as an important source of genomic variation in chickens and other livestock species, with potential relevance for adaptation and breed differentiation, but the functional consequences of individual CNVRs are frequently difficult to infer from positional overlap alone [8,17]. For DT, several CNVR-overlapping genes are plausibly connected to developmental regulation and tissue organization. Among these, *ZIC1* and *ZIC4* are notable because ZIC-family transcription factors are well-established regulators of embryogenesis and morphogenesis across vertebrates, including roles in developmental patterning and tissue specification. Several studies support the idea that ZIC genes function in multiple developmental contexts and may influence patterning trajectories through transcriptional regulation and interactions with signaling pathways [30,31,32]. In the context of the present study, the identification of *ZIC1/ZIC4* within DT CNVRs does not demonstrate a direct effect on limb traits; nevertheless, their known developmental roles make them reasonable candidates for future investigation in relation to breed-specific morphology.

Similarly, the presence of *JARID2* and *KMT2C* in DT CNVRs is intriguing because both genes encode chromatin-associated regulators implicated in developmental gene expression programs. *JARID2* is linked to epigenetic regulation and transcriptional control during development, including through interactions with chromatin regulatory complexes, whereas *KMT2C* and *MLL3* are histone methyltransferase components associated with enhancer regulation and broad transcriptional control. The developmental relevance of these genes is well supported in the literature, although much of the evidence derives from mammalian systems and disease-oriented contexts [33,34]. In the present dataset, their overlap with CNVRs is best interpreted as indicating that structural variation may affect regions containing epigenetic regulators with potentially pleiotropic functions. This raises the possibility of subtle effects on developmental robustness, tissue differentiation, or quantitative trait variation, but functional studies are required to test such hypotheses. Another DT-enriched functional theme concerns extracellular matrix biology and connective/skeletal tissue-related genes. The CNVR-overlapping set includes *PLOD2*, *OGN*, *OMD*, and *BGN*, all of which are associated with ECM organization, collagen architecture, mineralized tissue biology, or connective tissue function. *PLOD2* is involved in collagen lysine hydroxylation and cross-link formation, processes that are important for collagen maturation and tissue mechanical properties; disruptions in *LH2/PLOD2*-related pathways are linked to abnormal collagen cross-linking and skeletal/connective tissue phenotypes [35,36]. *OGN* and *OMD* are small leucine-rich proteoglycan family members implicated in ECM organization and bone-associated processes, and the literature supports roles for osteoglycin and osteomodulin in matrix biology, mineralization-related contexts, and osteogenic processes, though effects are context-dependent [37]. Taken together, the co-occurrence of multiple ECM/collagen-associated genes in DT CNVRs is compatible with the hypothesis that structural variation in this breed may involve genomic regions relevant to tissue architecture and biomechanical phenotypes. At the same time, this remains a correlative observation based on genomic overlap and should not be overinterpreted as direct evidence of altered skeletal development. In DT, the presence of *LRP4* within CNVRs is of potential interest because this gene has recognized roles in bone biology and skeletal homeostasis; however, in the present framework, it should be considered a candidate locus for follow-up rather than a confirmed determinant of morphology [38]. DT CNVRs also overlapped genes related to tissue structure/remodeling (*ACTA1*, *CREB3L2*) and to physiological adaptation, redox balance, and innate defense (*EGLN1*, *GPX1*, *OASL*, *DUOX1/DUOXA2*). Although these functions are biologically plausible in the context of local adaptation, the current evidence remains hypothesis-generating, since CNVR overlap alone does not demonstrate functional effects [39].

In RI and CC, genes such as *CACNA1S*, *CALCR*, *CAPN3*, and *MAPK13/MAPK14* support the presence of CNVRs in regions functionally related to muscle physiology, calcium/bone signaling, and stress/inflammatory pathways, but no direct inference on breed-specific functional divergence can be made without expression or association data. Overall, DT, CC and RI CNVRs intersect genes involved in multiple biological systems (developmental regulation, ECM/connective tissue biology, muscle function, redox homeostasis, and innate immunity), consistent with a complex and potentially multifactorial contribution of structural variation to local breed diversity. This interpretation is also in line with previous CNV studies in chicken [17]. The detection of CNVRs on chromosome 16 is consistent with previous reports, as this chromosome is often described as CNV-rich and contains the MHC, a key immune-related region. Nevertheless, CNV inference in MHC-rich regions should be interpreted cautiously because of sequence complexity and mappability-related limitations [17]. Overall, these results provide a prioritized set of candidate regions for future validation, rather than direct evidence of causal effects on breed-specific phenotypes.

The QTL overlap analysis further supports this interpretation. DT had a far greater number of QTL intersections compared with CC and RI, suggesting stronger colocalization of structural variants with genomic regions previously associated with bone growth, limb length, and overall skeletal robustness in chickens. Several enriched QTL intervals fall within chromosomal regions known to harbor quantitative determinants of tibia length, bone mineral density, and body size [40,41]. This convergence between CNV-driven structural variation and QTL linked to limb elongation underscores the likelihood that the DT phenotype results from multiple interacting genetic mechanisms. By contrast, the CC and RI breeds showed fewer CNVR-associated genes and fewer QTL overlaps, which may reflect their differing breeding histories and phenotypic profiles. CC, characterized by moderate body size and standard shank proportions, displayed CNVs mostly enriched for metabolic and immune-related genes, suggesting that selection may have prioritized traits unrelated to skeletal elaboration. RI, with its comparatively low number of CNV-embedded genes, exhibited a genomic architecture consistent with reduced structural variation, which may align with its more conserved morphology and lower phenotypic divergence. The absence of extensive limb-development regulators among CNV regions in these two breeds supports the notion that DT represents a unique case of structural genomic remodeling targeting hindlimb growth pathways.

Collectively, these findings provide a genomic framework explaining the exceptional shank enlargement of the DT chicken. Structural variation affecting HOX clusters, BMP/GDF pathways, and key limb regulators likely act synergistically with QTL associated with skeletal elongation to drive dramatic breed-specific hindlimb hypertrophy. This combination of CNV-driven gene modulation and QTL overlap highlights several candidate regions for functional validation and offers a biological basis for phenotypic divergence among chicken breeds.

### 4.3. GO Term Analysis of CNVR-Associated Functions

The pathways (Figure 4) are commonly implicated in physiological regulation, behavior, and adaptation, suggesting that CNVRs may influence key regulatory mechanisms in Vietnamese indigenous chicken breeds. The representation of immune-related pathways is consistent with the known adaptability and disease resilience of indigenous chickens and may highlight structural variants contributing to immune modulation. This pattern suggests that CNVRs in these populations may affect specific regulatory or sensory processes rather than broad genomic functions. Overall, the diversity of enriched pathways indicates that CNVRs contribute to a wide array of biological mechanisms, potentially influencing behavior, metabolism, immune response, and adaptation. These findings support the idea that structural variation plays a meaningful role in shaping breed-specific traits and may represent a valuable resource for future functional and genomic selection studies in indigenous chickens.

Gene Ontology analysis allowed us to explore the potential biological roles affected by structural variation in the three chicken breeds. In particular, the Molecular Function category (Figure 5A) highlighted essential molecular activities, indicating that CNVRs are not randomly distributed across the genome but are preferentially located in regions involved in fundamental regulatory and biochemical processes. The presence of several genes linked to catalytic and binding functions may reflect structural variation affecting metabolic flexibility and cellular responsiveness traits that often underlie adaptation to local environments. In Biological Process (Figure 5B), the categories indicate that CNVRs may influence pathways tied to physiology, adaptation, and immune response. It is particularly noteworthy that homeostasis and immune system processes were enriched, as these are traits that frequently differentiate indigenous breeds known for their resilience and disease tolerance. Protein Class enrichment (Figure 5C) suggests that CNVRs might affect gene networks directly involved in metabolism, cellular regulation, and signal transduction. The presence of structural proteins and scaffolding proteins hints at possible links between CNVRs and morphological traits within these breeds. Figure 5D (the Cellular Component category) reinforces the idea that CNVR-associated genes participate in core cellular structures and complexes rather than isolated or peripheral cellular components. Taken together, the GO enrichment results show that CNVRs in these three Vietnamese breeds are connected to a wide variety of biological functions, especially those governing metabolism, cellular regulation, signaling, and immune capacity. This diversity suggests that structural variation plays a meaningful role in shaping both the physiological and adaptive characteristics of these populations. These findings are consistent with the known traits of indigenous chickens, which often display strong adaptability, disease resistance, and distinctive phenotypic features shaped by both natural and human selection.

### 4.4. Breed Relationships Revealed by CNVRs

CNVR-based hierarchical clustering (Figure 6) does not support a fully breed-specific compact cluster for DT; instead, DT individuals are distributed across multiple branches, indicating that the breed includes both shared and heterogeneous CNVR patterns. This result is consistent with the distinctive morphology of DT chickens, especially their enlarged shanks [36], but suggests that the underlying genomic architecture is unlikely to be explained by a single uniform CNVR signature. Rather, DT appears to combine a partially shared structural genomic background with substantial intra-breed variation, in agreement with the high number of single-animal CNVRs detected earlier.

A notable feature of the dendrogram is the presence of mixed subclusters involving DT and CC individuals (e.g., the highlighted cluster containing DT_4, CC_4, DT_6, and CC_3), supported by relatively high AU *p*-values, as well as additional inter-breed associations in other branches. This pattern indicates partial overlap in CNVR profiles among breeds and suggests that CNVR-defined relationships do not strictly mirror breed labels. The intermixed clustering of RI and CC individuals, and more broadly the occurrence of cross-breed clustering throughout the tree, is consistent with the CNVR overlap results and may reflect shared ancestry, gene flow, or similar ecological and management conditions acting on these populations over time.

The moderate or low AU support observed for several internal branches is not unexpected, as indigenous breeds typically show substantial individual genomic variability [16,38,39], and CNVR-based clustering can be influenced by the sparse and heterogeneous nature of structural variants. Therefore, the clustering pattern should be interpreted as evidence of partial genomic structuring rather than clear-cut breed separation.

Taken together, these results suggest that CNVRs capture both within-breed heterogeneity and between-breed connectivity in Vietnamese indigenous chickens. In this framework, DT does not emerge as a uniformly distinct CNVR cluster, but rather as a breed with a complex structural genomic profile, combining internal diversity with partial sharing of CNVR patterns with CC (and, in some branches, RI). These findings further support the usefulness of CNVRs as informative markers of population genomic structure, while also highlighting the complexity of genomic diversity in indigenous chicken populations.

### 4.5. Limitations of the Study

Some limitations of the study are worth acknowledging as they also represent areas of improvement in future studies. First, the sequencing depth (3–5×) may have reduced the sensitivity for detecting small CNVs or complex structural rearrangements, potentially leading to false negatives in low-mappability genomic regions. Second, although two read-depth-based algorithms (FREEC and CNVpytor) were applied and stringent filtering criteria were used, breakpoint resolution remains limited at this sequencing depth. Third, experimental validation was performed on a limited number of CNVRs, which is common practice in genome-wide CNV studies but does not exclude the possibility of additional false positives. Finally, the study focuses on structural variation discovery and genomic characterization, and therefore, no direct association analyses with phenotypic traits were performed. Consequently, the biological interpretation of CNVR–gene or CNVR–QTL overlaps should be considered hypothesis-generating and requires further validation through functional and association studies in larger populations.

## 5. Conclusions

This study is the first analysis on the genomic distribution of CNVs/CNVRs in Vietnamese native DT, CC, and RI chickens using NGS technology. A total of 1743 CNVs, corresponding to 122 unique CNVRs, were identified across the three breeds and confirmed by qPCR. Our results also revealed 3633 unique genes overlapping with these CNVRs. DT showed the highest number of CNVR-embedded genes, consistent with a broader structural genomic variability in this breed. The results highlighted candidate genes and pathways potentially related to developmental regulation, extracellular matrix/connective tissue biology, physiological regulation, and immune/stress responses, rather than a single major developmental gene cluster. In addition, QTL overlap analysis identified CNVR colocalization with genomic regions previously associated with skeletal and morphometric traits, supporting the biological relevance of several candidate regions, while requiring further validation in larger populations and functional studies. These results provide a genomic baseline for future research, particularly genome-wide association studies (GWAS) in larger populations of local chicken breeds. A GWAS targeting the distinctive shank phenotype of DT chickens would allow the identification of genomic loci associated with this trait. In a subsequent step, transcriptomic analyses could be performed to profile gene expression in relevant tissues, and integration of genomic and transcriptomic data would help clarify whether candidate genes or loci identified by GWAS also show expression patterns consistent with their functional involvement, e.g., in shank development.

## Figures and Tables

**Table 1:** Descriptive statistics of copy number variation regions (CNVRs) identified in the Vietnamese chicken breeds.

Breed	*n*	CNVRs			Total *	Number of CNVs	Mean Length (bp)	Min Length (bp)	Max Length (bp)	Genome Coverage (%)
		Loss	Gain	Mixed						
Cay Cum	6	44	45	17	106	458	1,073,179	11,339	11,650,000	7.6
Dong Tao	12	58	48	23	129	855	949,798	15,789	1,036,667	8.6
Ri	6	56	6	18	80	430	1,069,126	21,523	10,366,599	4.7

* Counts include both breed-specific and shared CNVRs.

**Table 2:** Genome coverage (%) by chromosomal copy number variation regions (CNVRs), calculated using non-redundant CNVRs at the genome level.

Chromosome	Loss	Gain	Mixed	Total	Length	Coverage (%) *
1	3	9	3	15	7,933,800	4.0
2	10	8	1	19	9,550,000	6.4
3	3	3	1	7	4,172,700	3.8
4	4	5	0	9	4,100,000	4.5
5	8	1	0	9	5,976,900	10.0
6	2	3	1	6	4,150,000	11.5
7	3	0	0	3	1,900,000	5.2
8	5	0	0	5	4,200,000	14.2
9	3	0	0	3	4,616,900	19.5
10	3	2	1	6	3,193,700	15.6
11	2	1	0	3	3,600,000	18.3
12	3	2	0	5	2,850,000	14.2
13	1	1	1	3	2,000,000	11.2
14	2	1	1	4	4,040,200	26.4
15	0	0	1	1	779,400	6.1
16	0	1	1	2	2,332,600	82.0
17	1	0	0	1	1,100,000	9.9
18	0	2	1	3	1,599,400	13.8
19	0	0	1	1	1,600,000	8.2
20	1	0	1	2	4,300,000	30.1
21	0	1	0	1	350,000	5.0
22	0	3	0	3	700,000	14.9
23	0	0	1	1	1,020,200	16.3
24	1	1	0	2	2,641,900	40.8
25	1	3	0	4	1,350,000	44.0
26	0	1	1	2	1,650,000	30.8
27	0	0	1	1	1,750,000	33.5
28	0	0	1	1	3,600,000	66.2
Total	56	48	18	122		

* Coverage of CNVRs by chromosome relative to each chromosome length.

## Data Availability

The datasets generated and/or analyzed during the current study are available from the corresponding author upon reasonable request.

## Supplementary Materials

The following supporting information can be downloaded at: https://www.mdpi.com/article/10.3390/ani16071085/s1, Table S1: Primer Sequences Used for qPCR Validation; Table S2: qPCR Validation Results of Selected CNVRs; Table S3: CNVR support by breed, reporting the number of individuals carrying each CNVR in DT, CC, and RI; Table S4: List of Genes Overlapping CNVR Regions; Table S5: QTLs Mapped Within CNVRs; Table S6: Number of CNVRs Across Literature. References [5,8,9,11,12,13,14,15,16,17,18,19,20,21] are cited in the Supplementary Materials.

## Abbreviations

The following abbreviations are used in this manuscript:

ACTA1	Actin alpha 1
BGN	Biglycan
CACNA1S	Calcium voltage-gated channel subunit alpha1 S
CALCR	Calcitonin receptor
CAPN3	Calpain 3
CC	Cay Cum chicken
CNV	Copy number variant
CNVR	Copy number variation region
CREB3L2	cAMP responsive element binding protein 3 like 2
DT	Dong Tao chicken
DUOX	Dual oxidase
EGLN1	Egl-9 family hypoxia inducible factor 1
GPX1	Glutathione peroxidase 1
JARID	Jumonji and AT-rich interaction domain containing 2
KMT2C	Lysine methyltransferase 2C
LH2	LIM homeobox 2
LRP4	LDL receptor related protein 4
MAPK13	Mitogen-activated protein kinase 13
OASL	2′-5′-oligoadenylate synthetase like
OGN	Osteoglycin
OMD	Osteomodulin
PLOD2	Procollagen-lysine,2-oxoglutarate 5-dioxygenase 2
qPCR	Quantitative Polymerase Chain Reaction
QTL	Quantitative Trait Loci
RI	Ri chicken
ZIC	Zic family member
